# Re-inventing ancient human DNA

**DOI:** 10.1186/s13323-015-0020-4

**Published:** 2015-05-01

**Authors:** Michael Knapp, Carles Lalueza-Fox, Michael Hofreiter

**Affiliations:** Molecular Ecology and Fisheries Genetics Laboratory, School of Biological Sciences, Bangor University, 3rd Floor, Deiniol Road, Bangor, LL57 2UW UK; Department of Anatomy, University of Otago, 270 Great King St, Dunedin, 9016 New Zealand; Institute of Evolutionary Biology (CSIC-UPF), Doctor Aiguader, 88, 08003 Barcelona, Spain; Department of Mathematics and Natural Sciences, Evolutionary and Adaptive Genomics, Institute for Biochemistry and Biology, University of Potsdam, Karl-Liebknecht-Str. 24-25, 14476 Potsdam, Germany

**Keywords:** Archaic humans, Human evolution, Human population genomics, Next/second-generation sequencing

## Abstract

For a long time, the analysis of ancient human DNA represented one of the most controversial disciplines in an already controversial field of research. Scepticism in this field was only matched by the long-lasting controversy over the authenticity of ancient pathogen DNA. This ambiguous view on ancient human DNA had a dichotomous root. On the one hand, the interest in ancient human DNA is great because such studies touch on the history and evolution of our own species. On the other hand, because these studies are dealing with samples from our own species, results are easily compromised by contamination of the experiments with modern human DNA, which is ubiquitous in the environment. Consequently, some of the most disputed studies published - apart maybe from early reports on million year old dinosaur or amber DNA - reported DNA analyses from human subfossil remains. However, the development of so-called next- or second-generation sequencing (SGS) in 2005 and the technological advances associated with it have generated new confidence in the genetic study of ancient human remains. The ability to sequence shorter DNA fragments than with PCR amplification coupled to traditional Sanger sequencing, along with very high sequencing throughput have both reduced the risk of sequencing modern contamination and provided tools to evaluate the authenticity of DNA sequence data. The field is now rapidly developing, providing unprecedented insights into the evolution of our own species and past human population dynamics as well as the evolution and history of human pathogens and epidemics. Here, we review how recent technological improvements have rapidly transformed ancient human DNA research from a highly controversial subject to a central component of modern anthropological research. We also discuss potential future directions of ancient human DNA research.

## Review

### Introduction

Research on ancient human DNA has a very mixed history. Already the first sequence, the presumed cloning and partial sequencing of 3.4 kilobases (kb) of a 2,400-year-old Egyptian mummy [1] later turned out to be the result of contamination with modern human DNA [2]. Because DNA from modern humans is ubiquitous in the environment, including on archaeological and other samples [3-7], false positive results due to contamination with modern human DNA have plagued the analysis of ancient human DNA ever since the beginning of this field of research. Moreover, disagreement over when an ancient human DNA sequence should be considered authentic has, at least for a long time, led to a schism of the field with one group of researchers tending to believe most results and the second group, until recently, dismissing a large proportion of results from human ancient DNA studies as unreliable (see for example the discussions between Stoneking and Cooper [8,9] or [10,11]). For some time, researchers tried to assure authenticity of ancient human DNA sequences by following a more or less complete set of criteria including the use of a physically isolated work area, no-template control amplifications, reproducibility of experiments, cloning of PCR products, independent replication of key results in a second, independent laboratory, the evaluation of biochemical preservation of specimens, quantitation of the number of template molecules from which a PCR started, evaluation of fragment length distribution (‘appropriate molecular behaviour’: ancient DNA should be short) and the parallel analysis of non-human associated remains from the same site to evaluate sample contamination and DNA preservation. However, basically, all studies followed only some of these criteria, and there is no reason to assume that adherence to authenticity criteria could exclude contamination [12], as all criteria are of limited use when dealing with human samples that were contaminated during excavation or pre-laboratory handling, which is a common problem [13]. Thus, in order to convincingly ensure the authenticity of ancient human DNA sequence data, the key focus has shifted to avoiding contamination at excavation sites and, in the many cases when this cannot be achieved, to identifying contamination post hoc from the sequence data. These are the areas in which major improvements in recent years have greatly contributed to a new confidence in ancient human DNA research, resulting in the recent boom of human ancient DNA studies.

### Avoiding contamination during sample handling

The term ‘contamination’ in the context of DNA data from ancient human remains is used to describe several types of undesired DNA. This includes microbial DNA, which has become the centre of focus with the introduction of second-generation sequencing (SGS) and ancient genome sequencing. It is often abundant, reduces the percentage of endogenous DNA in ancient DNA extracts, and therefore increases the sequencing cost. Traditionally, though, the most problematic form of contamination of ancient human DNA is modern human DNA introduced during handling of samples prior to DNA sequencing. This second type of contamination will be the main subject of the following discussion.

During the first 10 or 15 years of ancient DNA research, the measures adopted to prevent contamination were focused on laboratory strategies. This was probably due to the fact that some obviously incorrect results (for example [14,15]) arose from carry-over contamination created in the laboratories themselves [16,17]. However, once a set of standard precautions was implemented, such as - among others - physical isolation of pre- and post-PCR areas, sterile material and gear, and restricted access to ancient DNA rooms (see for example [18]), it became increasingly evident that another form of contamination, the one that takes place before the samples reach the ancient DNA laboratories, is even more difficult to control. When skeletal remains are unearthed, handled and cleaned, the procedures applied often allow pervasive contamination of the samples with DNA of the people who have manipulated them. Since most European remains are excavated by ethnic Europeans, their DNA sequences may be closely related, if not indistinguishable, from those of the ancient specimens. This background human contamination was directly detected by analysing ancient animal samples, such as cave bear bones, in which the endogenous sequences are easily distinguishable from those that are contaminants [3,4,6]. During the last years, different ancient DNA studies have investigated the contamination process in detail [7,8,19,20], coming to a number of conclusions. (1) Samples are regularly contaminated by modern human DNA. (2) Although bones are more easily contaminated than teeth, both types of samples can be readily contaminated. (3) Beyond the visual evaluation of sample preservation and common sense with regard to the age and environment a sample comes from, there is no reliable method to evaluate DNA preservation in samples prior to the actual genetic analysis [21,22].

Therefore, the problems associated with contamination of samples during pre-laboratory treatment remain major challenges in ancient human DNA research. Studies have shown that contamination correlates with sample structural preservation and particularly with porosity of the sample [23]. External contamination is thereby most likely to be introduced at the time of first handling after excavation [13]. Using the information obtained from such studies on the origin of pre-laboratory contamination, field techniques can be improved to reduce the risk of contaminating samples [24]. Furthermore, raising awareness of the problem among excavators and introducing good practise guidelines can contribute to reducing the risk of sample contamination [25]. However, a large number of samples used for ancient human DNA studies are from remains that have been held in museums and extensively handled, often before DNA technology was even invented. To access genetic data from those samples, it is necessary to *a posteriori* evaluate the level of contamination in the sequence data, as *a priori* prevention of contamination is not possible for those samples.

### Recognizing contamination in DNA sequence data

#### DNA fragmentation

It has been suspected for a long time that endogenous and contaminant DNA may differ in length. The underlying idea is that because contaminants are much more recent than the endogenous sequences, it is expected that the chemical processes that fragment the DNA have had less time to operate. In one of the first studies that systematically investigated this question, undertaken with prehistoric dog and medieval cattle remains contaminated with human DNA [20], amplicons of different lengths were generated and sequenced. The authors observed that the ratio of authentic versus contaminant DNA increased as the PCR product length decreased (with the amplicon lengths ranging from 70 base pairs (bp) to 180 bp). However, massively parallel sequencing technologies have shown that in all Neanderthal samples studied so far, the two types of DNA molecules (endogenous and contaminants) overlap in size and are therefore indistinguishable from the fragment length distribution alone [26,27]. Hence, there is currently no evidence that endogenous and contaminating DNA could be distinguished based on molecule length alone. On the other hand, most endogenous ancient DNA fragments are small, with large proportions often under the 60 to 70 bp effective limit of PCR techniques [28]. Thus, the capacity of SGS technology to sequence shorter molecules than standard PCR and Sanger sequencing already dramatically reduces the risk of sequencing contaminants by fishing in a larger pool of endogenous molecules [29].

#### DNA deamination damage pattern

Prior to SGS technology, many research groups cloned PCR products to identify discrepancies between individual PCR amplified molecules. A common cause for such discrepancies in ancient DNA experiments is *post mortem* DNA damage [30,31]. DNA damage can be described as a set of lesion-induced substitutions caused by cytosine deamination events, producing C to T changes (or G to A if the damage took place on the opposite DNA strand [32]). It has been suggested some time ago that DNA damage patterns could be used for distinguishing endogenous from contaminant sequences and even that it may be possible to take advantage of the accumulation of DNA damage in ancient templates to estimate the probability of a particular sequence to be the original one, even if it is not present among the sequences obtained [33]. High throughput sequencing technologies have revealed an additional, previously unnoticed (and in fact unnoticeable with PCR) aspect of ancient DNA damage patterns, an increase of cytosine deaminations close to both ends of ancient DNA template molecules [34,35]. Detailed analysis of this pattern in many ancient samples has revealed that the frequency of this damage increases - with relatively large variation, probably due to the additional roles played by temperature [36] and other environmental burial conditions [37] - with increasing age of the sample [38] and may reach values >50% in very ancient samples [39,40]. Moreover, recent studies have shown that this damage pattern can be used to distinguish truly endogenous ancient sequence reads from contaminating sequences [40-42]. Using post-sequencing selection of reads carrying terminal C-T and G-A substitutions, it was possible to both reconstruct the most ancient hominin DNA sequence to date (a complete mitochondrial genome from a 400,000-year-old hominin fossil from Sima de los Huesos in Atapuerca [40]) and to retrieve an authentic mitochondrial genome sequence from a heavily contaminated Neanderthal sample [42].

#### Internal consistency of DNA sequence data

Compared to traditional PCR and cloning strategies, both SGS shotgun sequencing and target enrichment strategies coupled with SGS approaches can provide very high sequencing coverage of target regions combined with the possibility to sequence and distinguish a large number of individual template molecules. A further key improvement compared to early ancient human DNA studies is the availability of large amounts of whole genome reference data. By combining high coverage of target regions and haplotype information from modern human genomes, it has become possible to test DNA sequence data from ancient human remains for internal consistency. For example, hierarchical classifications of y-chromosomal and mitochondrial human haplotypes covering almost the entire present day human diversity are available. Therefore, every mutation characterising a haplogroup on the tree of human y-chromosomal or mitochondrial diversity is associated with known mutations along the branches of the tree leading to the respective group of sequences. Investigating mitochondrial genome or y-chromosome consensus sequences from ancient human samples, it is possible to target mutations along each branch of the tree and evaluate whether they all belong to the same haplotype. If this is the case, it increases the likelihood that all sequence data comes from a single biological source and is therefore less likely to be contamination.

At the autosomal level, it is possible to take advantage of linkage disequilibrium (LD) patterns; incompatibilities in fine-scale haplotypic structure - especially in highly variable regions - can provide direct estimates on the level of autosomal contamination [43]. Once the haplotype of an individual has been confirmed, individual sequencing reads covering mutations can be evaluated for consistency with this haplotype, thereby providing an estimate of the percentage of contaminating reads in the sequence data [44-46]. A further additional test, independent of the haplogroup attribution, would be to focus on those genetic variants found in the ancient specimen that are absent or at low frequency in a modern reference dataset [29]. However, this test only works for differentiated populations like humans and Neanderthals, but cannot be applied if, for example, Neolithic skeletons from Europe are studied genetically. Critically, these authentication strategies do not rely on samples that have been excavated under controlled conditions but can be applied to extensively handled museum samples, thereby greatly increasing the pool of human fossil remains available for genetic analyses.

#### Potential pitfalls of using SGS for sequencing ancient human DNA

While SGS technology has greatly improved our ability to identify contamination and other sequencing errors, the use of SGS technology is no guarantee against contamination. In fact, one of the earliest studies that applied SGS technology on human remains [47] was later criticized for potential contamination issues [48,49]. In this example, two research groups [47,50] sequenced genomic DNA from the same Neanderthal individual. The DNA extracts were produced following stringent ancient DNA authenticity criteria in a purpose-built cleanroom facility. One of the groups (Noonan *et al*. [50]) then used a standard cloning and Sanger sequencing approach to produce the sequence data, while the other one (Green *et al*. [47]) used SGS. Surprisingly, the results presented by the two groups differed significantly, with the data by Green *et al*. [47] showing evidence of modern human admixture in the Neanderthal population that was completely absent from the data presented by Noonan *et al*. [48-50]. Green *et al*. [27] later showed that the two extracts had left the cleanroom facility with very low levels of modern human contamination but that the Green *et al*. [47] extract was then contaminated with modern human DNA in the subsequent library preparation for SGS, which was conducted in a different, non-clean room laboratory.

In fact, some contamination risks associated with traditional PCR and Sanger sequencing studies are even increased by SGS technologies. This includes the cross contamination of experiments by PCR products from previous experiments. In contrast to non-cloned PCR products, SGS sequencing libraries are characterised by universal sequencing primer-annealing sites (‘adapters’) [51]. Furthermore, very high copy numbers of these sequencing libraries are often produced, for example for target enrichment approaches (see for example [52]). These amplified sequencing libraries may not only introduce human contamination from every part of the genome rather than just those parts amplified in previous studies, but they could also decrease the percentage of endogenous DNA in an ancient DNA sequencing library.

Finally, SGS technology itself can lead to erroneous results, for example through the potential misidentification of samples that were not sequenced individually but together with other samples. As a result of the very high throughput of SGS technologies, often more than one sample can be sequenced in the same sequencing run. Unique ‘barcodes’ (short stretches of unique DNA sequence incorporated in the sequencing adapters) are then used to separate sequencing reads from different samples. However, Kircher *et al*. [53] have shown that this can lead to sample misidentification by barcode cross-contamination and as a result of sequencing inaccuracy in the SGS process itself. They found that in order to avoid such issues, not only one, but both universal sequencing adapters have to be barcoded.

Thus, while eliminating or reducing well-known sources of erroneous sequence data from ancient human remains, SGS comes with its own set of challenges and pitfalls, which need to be taken into account when designing experiments and analysing sequencing data (see below).

### Re-inventing ancient human DNA

#### Major results of the PCR age

Despite limitations and criticism, there is no doubt that the use of standard PCR technology has provided significant insights into ancient human history and evolution. In 1997, Krings *et al*. [54] published the first mitochondrial DNA sequences from a Neanderthal, providing new insights into the relationship between anatomically modern humans and their closest, extinct relatives and starting the field of Neanderthal genetics. In 2005, Haak *et al*. [55] analysed 413 base pairs of mitochondrial control region from 24 early European farmers. They found that a mitochondrial haplotype common among Neolithic farmers some 7,500 years ago is rare in the modern European population and therefore suggested that early farmers had limited success in leaving their genetic mark on today’s female lineages in Europe. In a more recent study, Bollongino *et al*. [56] analysed a dataset of PCR amplified mitochondrial control regions from 25 Neolithic skeletons (supplemented by second-generation sequenced mitochondrial genomes from 6 of these individuals) from the Blätterhöhle in North West Germany. Combined with stable isotope analyses, the study revealed that Neolithic hunter-gatherers and farmers coexisted at the site for at least 2,000 years. Moreover, Bollongino *et al*. were able to show that in contrast to the early Neolithic farmers studied by Haak *et al*. [55], the late Neolithic farmers did leave a genetic mark on today’s central European populations and could in fact be considered ancestors of today’s Europeans.

In a further study investigating human population dynamics in Neolithic central Europe, Brandt *et al*. [57] reconstructed the development of mitochondrial genetic diversity from the Early Neolithic to the Early Bronze Age from a sample of 364 prehistoric central European individuals, including many previously published sequences. The data showed that shifts in mitochondrial diversity occurred contemporaneously with key cultural shifts in prehistoric central Europe.

Similarly, functional genetic studies of ancient human remains were already conducted in the pre-SGS era using PCR and Sanger sequencing. For example, Hummel *et al*. [58] investigated the frequency of the CCR5-Delta32 allele in a total of 99 ancient human individuals ranging from the Bronze Age to the nineteenth century. The allele confers resistance to HIV-1 infection and its frequency in Caucasian populations was hypothesized to have increased rapidly as a result of the medieval plague epidemics. Hummel *et al*. found no evidence for a change in frequency over the last 2,900 years, showing that, in contrast to prior hypotheses, the medieval plague epidemics did not influence the frequency of this mutation [58]. In 2007, Lalueza-Fox *et al*. [59] identified an inactivating mutation in the melanocortin 1 receptor (mc1r) of two Neanderthals, suggesting the presence of red haired individuals in the Neanderthal population. This study was somewhat unique in that the authors not only determined the DNA sequence of the ancient allele but also expressed the according protein and investigated its functional properties. In the same year, Burger *et al*. [60] typed two nuclear loci associated with lactase persistence in ten Neolithic, Mesolithic and medieval anatomically modern humans from central and eastern Europe. None of the Mesolithic and Neolithic individuals showed the lactose persistence genotype common among modern Europeans, while the medieval sample was heterozygous at one of the two loci tested and homozygous for the lactase persistence genotype at the second locus. Although limited in their interpretations by the small sample size, the authors suggested that lactose persistence only gained abundance over the last 8,000 years due to strong positive selection.

Common to all these studies, though, is the relatively small amount of DNA sequence data on which conclusions were based. Even if all data analysed indeed originates from the individuals studied, the small amount of sequence data obtained necessarily limits the conclusions that can be drawn.

#### The SGS ancient human DNA boom

SGS, with its characteristically large numbers of short sequencing reads, was quickly recognized as ideally suited for studying ancient DNA. The first SGS platform was presented in 2005 [51] and was almost immediately implemented in ancient DNA research. Within a few month of the introduction of SGS, Poinar *et al*. [61] published 13 million bp from the nuclear genome of the extinct woolly mammoth. When compared with the 27,000 bp of cave bear sequence [62] that represented the largest nuclear data set available from an extinct species in the pre-SGS era, the data set obtained by Poinar *et al*. [61] represented a 480× increase [63].

**Figure 1:**
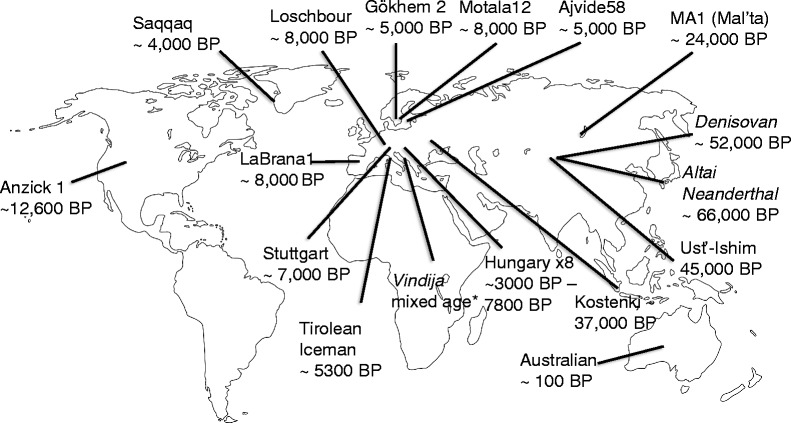
**Distribution and age of sequenced complete human genomes (>1× coverage).** Names in italics: archaic humans [64-77].

**Figure 2:**
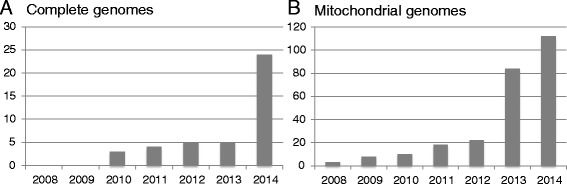
**Total number of published complete ancient human genomes (>1× coverage). (A)** Complete genomes. **(B)** Complete mitochondrial genomes. X-axis: year; Y-axis, total number of published genomes (cumulative). The genome of the Denisovan, which was sequenced to 1.9× coverage in 2010 and to 30× coverage in 2012, was only counted in 2010.

These improvements in sequencing technology have revolutionised, if not re-invented the field of ancient human DNA studies. In 2010, the first high coverage nuclear genome sequence from subfossil remains was obtained from a 4,000-year-old human hair tuft [64], bringing human ancient DNA to the technical forefront of ancient DNA research. The results allowed reconstructing the first human migration into Greenland and suggested that the ancestors of early Greenlanders migrated into the New World from Siberia some 5,500 years ago, independent of the migrations that gave rise to modern Native Americans and Inuit [64]. Since then, major breakthroughs in the study of human evolution and prehistory, including the analyses of complete genomes from key times and geographical locations in human history, have been announced on a regular basis (Figures 1 and 2A).

Also in 2010, the first low coverage Neanderthal draft genome was published [65]. In contrast to earlier mitochondrial genome studies such as the publication of the first Neanderthal DNA sequences by Krings *et al*. [54], the comparison of modern human and Neanderthal genomes provided evidence of admixture between Neanderthals and modern humans outside sub-Saharan Africa. In the same year, the ‘Denisovans’ became the first extinct group of hominins that was described almost entirely from DNA sequence data, in this case the complete mitochondrial genome [78], followed by a 1.9-fold coverage nuclear genome later the same year [79]. Two years later, the complete genome of the same Denisovan individual was sequenced to high coverage (30-fold; Meyer *et al*. [68]). In 2014, Prüfer *et al*. [73] also published a 52-fold coverage Neanderthal genome from an individual from the same Altai Mountains cave as the Denisovans. By comparing this Neanderthal genome to the high coverage Denisovan genome as well as 25 genomes from modern humans and two further low coverage Neanderthal genomes, they were able to confirm gene flow between all three groups of hominins and also found evidence of gene flow from an unknown fourth hominin group into Denisovans. Moreover, a comparison of modern human genomes to the newly obtained archaic hominin and great ape genomes allowed identifying a catalogue of mutations unique to modern humans.

#### Modern human population history in the light of ancient DNA

Interestingly, most high-throughput DNA sequencing studies on ancient humans to date have focused on obtaining large amounts of sequence data from single or very few individuals rather than targeting representative multilocus DNA sequence data from a large number of individuals, as is common practice for modern populations (for example [80-82]). The human genome reference database is now so extensive that even low coverage single ancient genomes can provide new insights into human population history. For example, in 2012, two separate studies [41,83] reported a total of only 307 Mb from two Mesolithic foragers, three Neolithic hunter-gatherers and a single farmer. Despite the comparatively small amount of data, both studies were able to show that hunter-gatherers are genetically more closely related to modern day northern Europeans. Furthermore, the study by Skoglund *et al*. [41] was able to demonstrate that the single Neolithic farmer individual showed a close genetic affinity to modern Sardinians. The later affiliation was also recovered with the 7.4× coverage genome of the Tyrolean ice man [67] and several other Neolithic individuals [84], suggesting that Sardinians represent to some extent a Neolithic relict population.

While most ancient human genome data reported to date originate from European specimens, researchers have started exploring the population history of other populations as well (Figure 1). Thus, in January 2014, Raghavan *et al*. [70] presented the genome of an approximately 24,000-year-old individual from Mal’ta in south-central Siberia, sequenced to an average coverage of 1×. Despite the low coverage, the genome provided evidence that Native Americans share a dual ancestry influenced by genetic contributions from both eastern Asian and western Asian populations. These results confirmed and expanded on earlier results based on modern genome data [85] which showed a signal of admixture into Northern Europe consisting of ancestral links to present day Basques and Sardinians as well as the northeast Asian/American component identified by Raghavan *et al*. [70]. While at first sight it may seem surprising that low coverage genome data can provide such insights with any level of confidence, it becomes more understandable when the total number of informative mutations used in these analyses is considered. Any one mutation characterising the ancestry of an ancient individual sequenced to low coverage may be a result of sequencing error, but the study described above compared 66,285 single nucleotide polymorphisms (SNPs) to a reference panel of 1,301 individuals. Given the large number of markers characterising the ancestry of the individual, the chances that sequencing errors at known SNP sites alone result in incorrect ancestry inferences are therefore comparatively small. In a similar study, Rasmussen *et al*. reported a 14× coverage genome of an approximately 12,500-year-old North American member of the Clovis culture. This study provided evidence that the Clovis people are the direct ancestors of present day Native Americans, a question that had been the subject of a long-term controversy. Finally, the low coverage genome of a 7,000-year-old Mesolithic European from northern Spain provided a first glimpse into the phenotype of early European hunter-gatherers by revealing a combination of relatively dark skin and blue eyes [69]. In summary, the trickling of recent individual ancient genome studies leaves no doubt about the usefulness of whole genome data for gaining insights into the history and origin of present day populations or even phenotypes.

**Figure 3:**
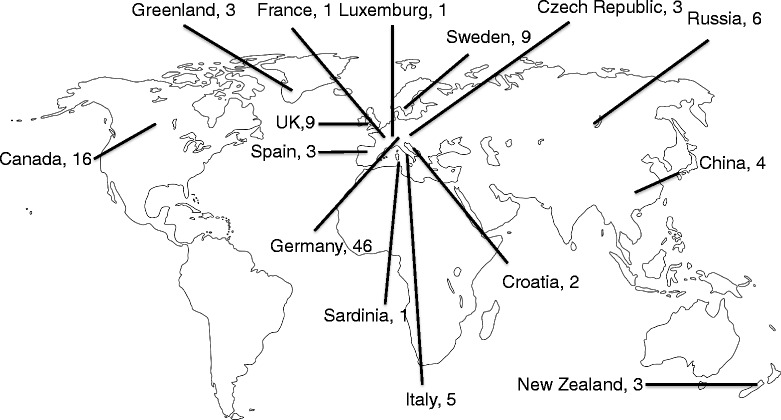
**Distribution and number of ancient human mitochondrial genomes sequenced to at least 1× coverage.** Only those mitochondrial genomes that were published independently from the complete genome of the respective individual were counted [26,29,40,45,46,56,71,78,83,86-96].

Despite these successes, limited sample numbers will always limit the power of conclusions and any extrapolation from such studies to entire populations has to be interpreted with caution. However, given the remarkable rate at which complete genome data (Figures 1 and 2A) or a combination of complete mitochondrial genomes and nuclear data from ancient human remains is now becoming available (Figures 2B and 3) [71,86,87], the small number of genome-sequenced ancient individuals is unlikely to be a limitation for much longer. The increasing number of complete ancient human genomes has for example already enabled a high-resolution analysis of the ancestry of Europeans [72]. In their study, Lazaridis *et al*. [72] found that present day Europeans derived from at least three highly differentiated ancestral populations, including west European hunter-gatherers, ancient north Eurasians, and early European farmers. It is important to note, though, that the still relatively small sample number (15 complete or partial genomes) limits the conclusions drawn from this - for the moment - comparatively large-scale ancient population genomic study. Only an increase in sample size will show whether the three ancestral populations identified represent indeed all sources of modern European diversity, or whether they are an artefact resulting from having analysed only a small number of samples from a limited geographical region.

#### Extending the range of ancient human DNA studies further into the past

Prüfer *et al*. [73] showed that by comparing the genomes of anatomically modern humans to their closest relatives, it is possible to identify mutations in the human genome that are unique to anatomically modern humans. If bone material and sequence data from more Neanderthal and Denisovan individuals did become available, similar studies may also become possible for these archaic humans. Using these data as basis, studies on how anatomically modern humans, Neanderthals and Denisovans differ on a functional genomic level will become possible. These studies will provide key insights into the evolution and divergence of all three human groups. However, to complete the puzzle of human evolution, information about the genome of the population ancestral to anatomically modern humans, Neanderthals and Denisovans would be essential. As this population existed more than 500,000 years ago [73], this was until recently considered an improbable challenge. However, using improved DNA extraction techniques [39] along with a highly sensitive SGS library preparation protocol [97], Meyer *et al*. [40] were able to sequence the complete mitochondrial genome of a 400,000-year-old hominin from Sima de los Huesos cave in Atapuerca (Spain). As mitochondrial DNA alone has been shown to be an unreliable source of phylogenetic information in hominins [68,78,79], it was not possible to draw firm conclusions about the role of the Sima de los Huesos hominin in human evolution. Nevertheless, the study provides an important first step towards the analysis of Middle Pleistocene hominin remains and raises the hope that ancient DNA may soon allow us to trace a substantial part of human evolution on the molecular level and in real-time.

## Conclusions

Ancient human DNA research today promises exciting insights into the evolution and history of our own species. However, despite major technological advances associated with SGS, authentication of ancient human DNA sequence data is anything but trivial. New guidelines for ensuring sequence data authenticity are required to deal with the fundamental changes in sequencing strategies introduced by SGS. Given such precautions, though, ancient human DNA research is likely to soon complete the transition from an interesting but marginal discipline of human evolution and history to a key component of anthropological research.

### Guidelines for SGS work with ancient DNA

These are not intended to represent strict ‘criteria’ that all need to be followed in any experiment, but rather recommendations to consider during experimental setup. Also, it is important to note that while the below measures reduce the probability to retrieve contaminating sequences and increase the probability to recognize contamination in case it has occurred, they are by no means a guarantee for contamination-free data sets. Rather, as noted before [12], they should be seen as help for researchers, reviewers and readers to critically evaluate SGS data obtained from ancient human samples. It is also important to emphasize that different studies will require different levels of authentication and, as has long been known (but very often ignored), the more unlikely data are - either from a technical or biological perspective - the more evidence of their authenticity is required. Finally, we would like to point out that the extreme sensitivity of SGS technology due to the extremely high number of reads obtained (up to several billions) makes it virtually impossible to obtain completely contamination-free data sets. Therefore, the question is no longer whether contamination (of the samples, the chemicals, plastic wear or the experiments) has occurred, but rather whether the contamination is severe enough to influence any conclusions drawn from the data.

1. Perform all molecular work from DNA extraction to library preparation (though not amplification!) in a dedicated ancient DNA lab. Follow a strict one-way policy for ancient DNA work, that is, once somebody has been in a laboratory where high-quantity DNA (modern DNA, PCR products or amplified libraries) has been handled, the person must not go back to the DNA lab the same day. This recommendation has been criticized based on the theoretical claim that DNA will persist on people’s hands over night, but in our and the experience of many of our colleagues, empirically this measure is highly effective against contamination, while violation of the one-way policy readily results in abundant contamination.
2. Perform blank extractions and blank libraries. The inclusion of blanks in ancient SGS studies is one of the most neglected measures that was standard for ancient DNA work in the PCR era. There may be some problems that libraries from blanks may produce excessive adapter dimers (especially when barcoding libraries, see below), making sequencing on the same lane as sample libraries difficult, but such libraries can be size-selected to remove adapter dimers before sequencing. Inclusion of blank libraries would almost certainly have uncovered the heavy contamination in the first SGS study investigating human fossils [47].
3. Measurable cytosine deamination damage patterns in the sequences especially at the ends of template molecules [34,35]. This type of damage has been shown to increase with age [38], so sequences obtained from tens of thousands of years old human samples not showing such damage patterns should be considered highly suspicious.
4. Internal consistency of all sequence data. This is especially easy for haploid sequences such as mitochondrial DNA and - in males - Y- and X-chromosomes. These sequences should not show any evidence of polymorphic positions (at least not above the background level of ancient DNA damage and sequencing errors). Moreover, due to the clonal nature and therefore lineage-like inheritance of mitochondrial DNA and Y-chromosomes, it is possible to test whether all positions variable in an ancient sample compared to modern sequences are consistent with the known mitochondrial or Y-chromosomal tree. Finally, in samples known from coverage information of X-chromosome vs. autosomes to be female, it is possible to estimate contamination from male sources by searching for fragments mapping to the non-recombining part of the Y-chromosome. The situation is more complicated for autosomal data, but human variability has been studied thoroughly enough that data sets can be studied for incompatibility of neighbouring SNPs (that is, physically close SNPs may be so tightly linked that it is highly unlikely that an individual homozygous at one position would be heterozygous at a linked position). Statistical evaluation of such patterns also allows estimating contamination levels.
5. PCR amplification of a short (~60 bp) piece of human DNA from extraction and library blank to evaluate levels of low-quantity contamination with modern human DNA. Also, it is possible to perform population analyses with short and long sequence fragments separately and also with 3′ and 5′ deaminated and non-deaminated fragments separately [41] to evaluate if sequence data may originate from two populations of templates going back to different sources.
6. Barcoding of all libraries, ideally uniquely, even when not planning to multiplex sequence, because these libraries could come back to haunt following experiments. This is especially useful when planning to ever perform capture experiments, as libraries before capture are very highly concentrated PCR products, which will contaminate every room they are opened in. After capture, however, the target is back to a DNA concentration. But as the capture product tube is opened, all the super high concentration non-target also gets released, and that may contain human DNA from none target regions, which will be a contaminant with perfect library adapters if these regions ever get targeted.
7. Sensible setup of experimental workflow also outside the ancient DNA lab. For example, capture experiments can easily get contaminated with non-target DNA or DNA from previous experiments if elution of the low-quantity captured products is performed in the same laboratory as washing of the capture arrays or beads to remove the high-quantity amplified non-target DNA. A simple solution to this is to perform elution and any handling of eluted target DNA prior to potential re-amplification in a dedicated laboratory.
8. Biological and technical sense. Although this is a difficult category and it is important that unexpected results are not *a priori* dismissed as contamination as this would prevent any scientific progress, it remains true that extraordinary results - from a technical or biological perspective - require also extraordinary evidence supporting them. However, this is also not to say that expected results have to be true. They also require supporting evidence.

## Abbreviations

A: adenine
bp: base pairs
C: cytosine
DNA: deoxyribonucleic acid
G: guanine
kb: kilobases
PCR: polymerase chain reaction
SGS: second-generation sequencing
SNP: single nucleotide polymorphism
T: thymine
